# Association between smokeless tobacco use and oral cavity cancer risk in women compared with men: a systematic review and meta-analysis

**DOI:** 10.1186/s12885-021-08691-x

**Published:** 2021-08-26

**Authors:** Guangyan Mu, Jiayi Wang, Zhiyan Liu, Hanxu Zhang, Shuang Zhou, Qian Xiang, Yimin Cui

**Affiliations:** 1 Department of Pharmacy, Peking University First Hospital, No. 6, Dahongluochang Street, Xicheng District, Beijing, 100034 China; 2 School of Pharmaceutical Sciences, Peking University Health Science Center, Beijing, China; 3 Institute of Clinical Pharmacology, Peking University, Beijing, China

**Keywords:** Smokeless tobacco, Oral cavity cancer, Sex-based difference, Meta-analysis

## Abstract

**Background:**

The impact of smokeless tobacco (SLT) use on the risk of oral cavity cancer (OCC) has been confirmed; however, the sex-based difference in this association remains inconclusive. Therefore, this study aimed to estimate the association between SLT use and OCC risk in women and compared it to that in men.

**Methods:**

PubMed, Embase, and Cochrane Library databases were systematically searched for eligible studies from their inception up to August 2020. Studies reporting the effect estimates of SLT use on OCC risk in men and women, were eligible for inclusion. The relative risk ratio (RRR) was applied to calculate the sex-based difference in the relationship between SLT use and OCC risk, and pooled analysis was conducted using a random-effects model with inverse variance weighting.

**Results:**

Nineteen studies reporting a total of 6593 OCC cases were included in the final meta-analysis. The pooled relative risk (RR) suggested that SLT use was associated with an increased risk of OCC in both men (RR, 2.94; 95% confidence interval [CI], 2.05–4.20; *P* < 0.001) and women (RR, 6.39; 95%CI, 3.16–12.93; *P* < 0.001). Moreover, the SLT-use-related risk of OCC was higher in women than that in men (RRR,1.79; 95%C, 1.21–2.64; *P* = 0.003). The risk of OCC related to SLT use in women was still significantly higher than that in men (RRR, 1.75; 95%CI, 1.15–2.66; *P* = 0.008) after excluding indirect comparison results. Finally, a subgroup analysis suggested significant sex-based differences only in individuals who received chewed smokeless products, regardless of the control definition. Pooled analysis of studies with high design quality confirmed the notably higher risk of OCC in women than in men.

**Conclusions:**

This study found that SLT use was associated with a higher risk of OCC in women than in men. Further large-scale prospective cohort studies should be conducted to verify sex-based differences in the association between use of specific smokeless products and OCC risk.

## Background

Oral cavity cancer (OCC) is the subtype of head and neck cancer and defined as any cancerous tissue growth in the oral cavity. It is the sixth most common cancer and accounts for nearly 4–5% of all cancer cases [1]. A total of 657,000 new cases of OCC occur annually, causing 330,000 deaths worldwide [2]. The prevalence of OCC is relatively high in some Asia-Pacific countries, especially in Taiwan, China, where the incidence rate reaches 32.46 per 100,000 persons [3–5]. The progression of OCC is complex, multistage and affected by both genetic and environmental factors, including human papillomavirus infection, smoking, and alcohol consumption [6, 7]. The 5-year survival rate of OCC ranges from 39 to 84% depending on the disease stage and from 48 to 67% for individuals of various ethnicities [8].

Smokeless tobacco (SLT) is marketed for oral (chewed, sucked, dipped, held in the mouth, etc.) or nasal use and contains different amounts of nicotine and nitrosamines [9]. SLT products, which are manufactured, stored, and consumed in many different ways, are used worldwide [10]. The mode of SLT use and the main ingredients vary based on geographic location, ingredient availability, cultural/societal norms, and personal preferences [11]. The use of SLT has already been illustrated as independent risk factor for OCC in numerous studies [12–14]. Ingredients of SLT products such as nitrosamines peculiar to tobacco, polycyclic aromatic hydrocarbons, nicotine, aldehydes and metals can form DNA adducts that induce oxidative damage and disrupt the cell growth cycle and further play a carcinogenic role on OCC [15, 16].

Tobacco chewing appears to be a major risk factor for oral and pharyngeal cancer in Asia [17]; however, the risk is not considered to be substantial among users of SLT products in the United States or Europe [18]. The difference in risk between Western countries and developing countries may be attributed to tobacco species, fermentation and aging [19]. According to data from the Global Burden of Disease, the overall incidence rate of OCC was higher in men than that in women, while women exhibited larger change trends than that demonstrated by men [20]. However, the sex-based difference in SLT-use-related OCC risk has not been well illustrated. Therefore, this systematic review and meta-analysis was conducted to illustrate the sex-based difference in the association between SLT use and OCC risk based on available studies reporting sex-specific effects.

## Methods

### Search strategy and selection criteria

This systematic review and meta-analysis was performed and reported following the Meta-analysis Of Observational Studies in Epidemiology (MOOSE) protocol [21]. The electronic databases of PubMed, Embase, and Cochrane Library were systematically searched for eligible studies from their inception up to August 2020.The following search terms were used: (“smokeless tobacco” OR “oral tobacco” OR “non burn tobacco” OR “snus” OR “gutkha” OR “naswar” OR “chew* tobacco” OR “tobacco powder” OR “tobacco tooth powder” OR “tobacco paste” OR “creamy snuff” OR “mishri” OR “masheri” OR “dip tobacco” OR “tobacco water” OR “tuibur” OR “hidakphu” OR “gul” OR “gutkha” OR “mawa” OR “khaini” OR “snuff” OR “pan masala” OR “pan masala with tobacco” OR “paan” OR “pan with tobacco” OR “zarda” OR “tambaku” OR “betel quid tobacco” OR “betel tobacco” OR “tobacco flakes” OR “tobacco leaf” OR “dried tobacco” OR “hogesoppu” OR “gnudi” OR “kadapa” OR “Mainpuri tobacco” OR “qiwam” OR “kimam” OR “dohra” OR “raw tobacco”) AND (“oral cancer” OR “oral carcinoma*” OR “oral malignant*” OR “oral tumour”). Studies reporting sex-specific relationship between SLT use and OCC risk were included. Both oral tobacco and tobacco that consumers did not smoke were included as SLT in our search strategy. No restrictions were placed on publication language and status. The references of the searched literature were also reviewed manually to further identify other eligible studies.

Two reviewers independently conducted the literature search and study selection following a standardized protocol. Discrepancies were settled by group discussion until a consensus was reached. The details regarding study inclusion criteria were as follows: (1) Participants: general population for cohort design, and OCC cases and non-cases for case-control design; (2) Exposure: SLT use; (3) Outcome: the prevalence of OCC and sex-specific effects of the relationship between SLT use and OCC risk; and (4) Study design: cohort, case-control, or case-reference studies.

OCC was defined by International Statistical Classification of Diseases and Related Health Problems 10th Revision (ICD-10) codes to distinguish the anatomic grouping and etiology of the disease. The studies that were included reported on cancers according with the following ICD-10 codes: C00-C06 and C09-C10, which included cancers of the lip, tongue, gum, floor of mouth, palate, cheek, vestibule of mouth, retromolar area, tonsil or oropharynx [22].

### Data collection and quality assessment

The following details of the included studies were independently extracted by two reviewers: first author, publication year, region (country in which the subject of the original study was located), study design, sample size (case/non-case), age and sex of participants, case definition, control definition, type of SLT product, confounders adjusted, matching of control, and reported sex-specific effect estimate. The Newcastle-Ottawa Scale (NOS) was used to assess the quality of observational studies, and this assessment was performed by two reviewers independently [23]. A study with 7 or more stars was considered to be of high quality, and those with 4–6 stars were regarded as moderate quality studies. Inconsistency in assessment regarding data collection and quality assessment were resolved by an additional reviewer by referring to the full text of the original article.

### Statistical analysis

The association between SLT use and OCC risk was assessed using a sex-specific effect estimates with a 95% confidence interval (CI). Given the low incidence of OCC, the odds ratio (OR) was approximately equal to the relative risk (RR). A random-effects model was applied to calculate pooled RRs and 95%CIs for the relationship between SLT use and OCC risk in men and women. The female-to-male ratio of RRs (RRRs) and 95%CIs were calculated using studies that reported the direct comparisons between men and women in terms of SLT use and OCC risk. The RRRs for indirect comparisons were calculated using the studies that only reported the relationship between SLT use and OCC risk in men or women. The pooled RRRs and 95%CIs for sex-based difference in the association between SLT use and OCC risk were calculated using a random-effects model [24]. Heterogeneity across the included studies was assessed using *I*^2^ and Q statistics, and *I*^2^ > 50.0% or *P* < 0.10 was considered to indicate significant heterogeneity [25].

Sensitivity analysis was conducted to assess the stability and reliability of the meta-analysis by excluding indirect comparison results [26]. Subgroup analysis based on direct comparison results was also performed according to the type of SLT product, control definition, confounders, matching of control, and study quality. Publication bias of all study arms was calculated using funnel plot, and Egger [27] and Begg [28] test results. The *P* values for the pooled results were two-sided, and the inspection level was 0.05. All statistical analyses were conducted using software the STATA software (version 15.1; Stata Corporation, College Station, TX, USA).

## Results

### Literature search and baseline characteristics

**Fig. 1:**
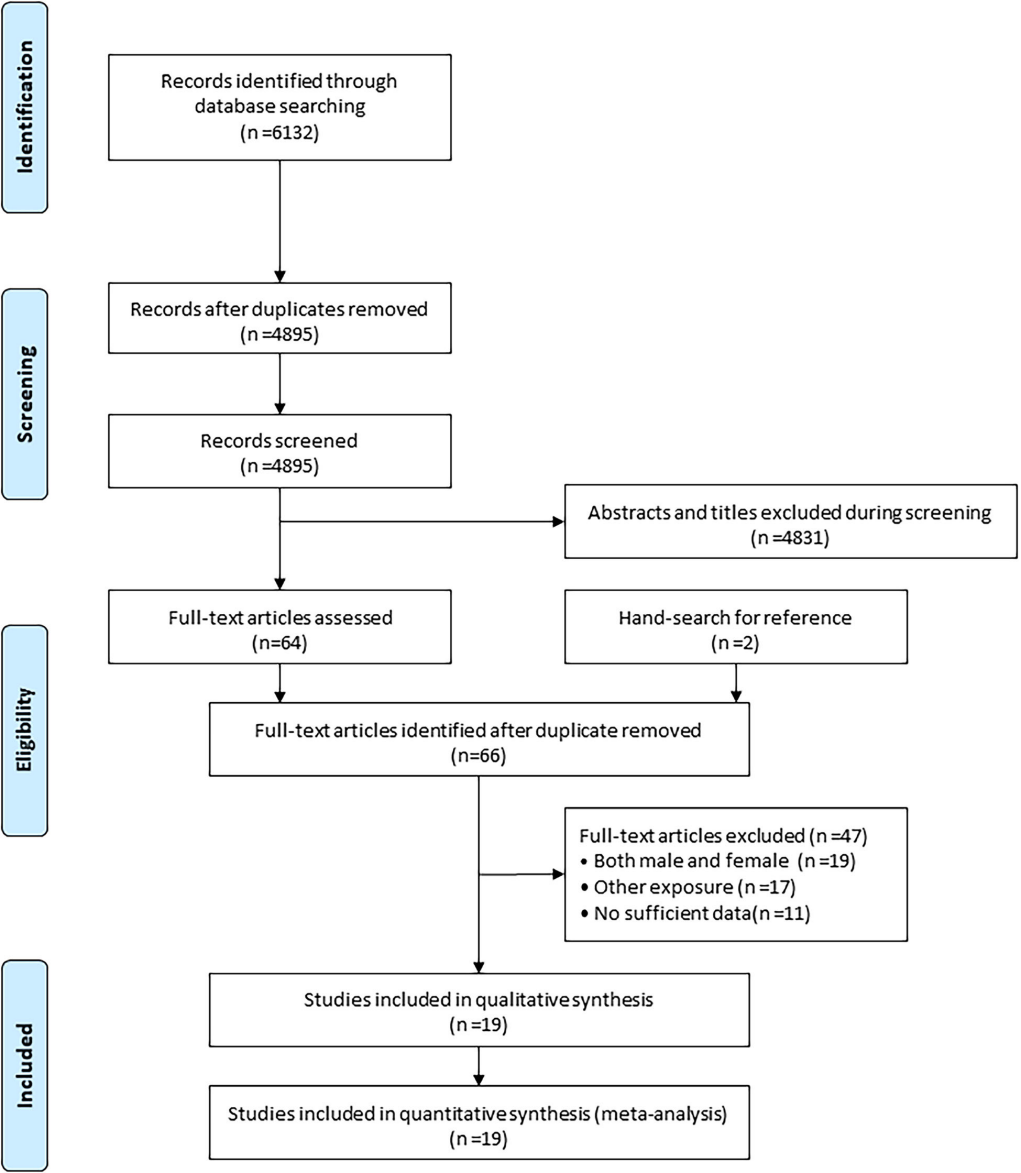
PRISMA flowchart for literature search and study selection

The electronic search yielded 6132 records, and 4895 articles were retained after duplicate removal. A total of 4831 articles were excluded after reviewing the title and abstract. The remaining 64 studies were retrieved for full-text evaluations, and 2 studies were obtained by manually searching the reference lists of the 64 studies. Thereafter, 47 studies were excluded for the following reasons: effect estimates were only provided for men and women combined (*n* = 19), other exposures were investigated (*n* = 17), or insufficient data (*n* = 11). Finally, 19 studies were selected for the meta-analysis [29–47] (Fig. 1).

The baseline characteristics of the included studies and participants are summarized in Table 1. The 19 included studies contained 6593 OCC cases (ranging from 84 to 1401). Fifteen studies were case-control or case-reference studies, and the remaining 4 studies were cohort studies. Thirteen studies were conducted in India, 2 in Pakistan, 2 in Sweden, 1 in the United States, and 1 study in Central and Southeast Asia. The sample size for individual studies ranged from 258 to 279,897. Seven studies had a NOS score ≥ 7 stars, and the remaining 12 studies had 5 or 6 stars.

**Table 1:** The characteristics of included studies and involved individuals

Study	Region	Study design	Sample size (case/non-case)	Age (yrs)	Sex (%)	Cases definition	Control definition	Type of smokeless product	Confounders adjusted	Matching of control	Study quality (NOS score)
Hirayama 1966 [29]	Central and south east Asia	Case-control	291 (173/118)	> 19.0	Male and female	Hospital cases of oral cancer	Non cancer cases from hospital	*Pan* tobacco, Manpuri, and other (Chew)	Restricted to non smokers	Age and sex	5
Winn 1981 [30]	US	Case-control	643 (233/410)	All stages	Female	Hospital cases of oral cancer	Non cancer cases from hospital	Oral snuff (NonChew)	Analysis of non smokers	Age, race, source of ascertainment, and country	6
Sankaranarayanan 1989a [31]	India	Case-control	681 (228/453)	All stages	Male and female	Histologically confirmed cases of tongue and floor of mouth from HBCR	Two non cancer for each case from hospital	*Pan* tobacco chewer (Chew)	Restricted to non smoker among man and non smoking non alcoholic among women	Age, sex and religion	7
Sankaranarayanan 1989b [32]	India	Case-control	1082 (187/895)	All stages	Male and female	Cancer Cases of gingiva from PBCR	Non cancer cases from hospital	*Pan* tobacco/ (Chew) Snuff (Non Chew)	*Bidi* alcohol and other Tobacco type	Unmatched	5
Nandakumar 1990 [33]	India	Case-control	696 (348/348)	55.0	Male and female	Histologically confirmed cases registered in PBCR for lip, tongue and mouth	Non cancer cases from hospital	Mixed for Tobacco, *Pan* and snuff (Chew)	Restricted to Non Smoking	Sex, age gp and residential area	6
Sankaranarayanan 1990 [34]	India	Case-control	1309 (414/895)	All stages	Male and female	Cancer Cases of buccal mucosa and labial mucosa registered in Trivandrum HBCR (1983–84)	Non cancer cases from hospital	*Pan* tobacco/(Chew) Snuff (Non Chew)	Bidi, alcohol And other tobacco type	Unmatched	6
Rao 1994 [35]	India	Case-control	1348 (713/635)	47.9	Male	Histologically confirmed oral cancer cases from hospital	Non cancer cases from hospital	Mixed *Pan*, betel nut, lime tobacco (Chew)	Analysis in non smoker and non alcoholic, stratification for age and residence	Unmatched	6
Lewin 1998 [36]	Sweden	Population based case referral oral cavity	653 (103/550)	40.0–79.0	Male	Incident Case from PBCR	Population based controls	Oral moist snuff (Non Chew)	Age, region, smoking and alcohol	Age group and region	7
Dikshit 2000 [37]	India	Case-control	408 (148/260)	All stages	Male	From PBCR records	Population controls randomly selected	Tobacco quid (Chew)	Age and smoking	Unmatched	6
Balaram 2002 [38]	India	Case-control	1173 (591/582)	18.0–87.0	Male and female	Histologically confirmed cases cases of oral cancer	Non cancer cases from hospital	*Pan* tobacco (Chew)	Age centre education chewing and (men only) smoking and drinking habits	Frequency matched control by centre quinquennium of age and gender	6
Znaor 2003 [39]	India	Case-control	1879 (281/1598)	≥ 25.0	Male	Histologically confirmed cases cases of oral cancer	Non cancer cases from hospital	Tobacco type not specified (Chew)	Age, centre, education level and smoking and alcohol drinking center	Sex	6
Luo 2007 [40]	Sweden	Retrospective cohort	279,897 (248/279,649)	≥ 35.0	Male	Users of snus From 1969 through 1992	Non users of snus	Swedish moist snuff (snus) (Non Chew)	Smoking	Not applicable	7
Muwonge 2008 [41]	India	Nested case-control	1692 (282/1410)	≥ 35.0	Male and female	Histologically confirmed Case that are newly	Randomly selected five non cancers from screened population	*Pan* with tobacco (Chew) Areca nut /lime +tobacco (Chew)	Smoking, alcohol education, religion	Sex, age panchayats and response status	7
Jayalekshmi 2009 [42]	India	Prospective cohort	78,140 (92/78,062)	30.0–85.0	Female	Former and current chewer females	Never chewer	Tobacco type not specified (Chew)	Age, family income, alcohol restricted to Non smokers	Not applicable	8
Pednekar 2011 [43]	India	Prospective cohort	87,222 (423/86,799)	≥ 35.0	Male	Male from voter list, cancer free at start. Patients registered in 150 government and private hospitals and institutions	Non user of SLT	Tobacco type not specified (Chew)	Age, education, religion, mother tongue, tobacco and BMI	Not applicable	8
Jayalekshmi 2011 [44]	India	Prospective cohort	66,277 (160/66,117)	30.0–84.0	Male	Former and current chewer females	Never chewer	Tobacco type not specified (Chew)	Age, calendar time, income and education	Not applicable	8
Ray 2013 [45]	India	Case-control	1250 (484/766)	10.0–99.0	Male and female	Histologically confirmed cases of oral cancer	Non cancer cases from hospital	*(Betal + gudhaka*) (Chew) Oral Snuff (Non Chew)	Restricted to Non Smokers and Non alcoholic	Area of residence	6
Arain 2015 [46]	Pakistan	Case-control	7556 (1401/6155)	30.0–60.0	Male and female	Histologically confirmed cases of oral cancer	Non cancer cases from hospital	*Gutkha* (Chew) NasalSnuff/(NonChew) *Manipuri* (Chew)	Smoking and Alcohol Other smokeless tobacco products (Restricted to non smoking and non alcoholic)	Age group, socio economic status and dietary habits	6
Khan 2017 [47]	Pakistan	Case-control	258 (84/174)	57.0	Male and female	Histologically confirmed cases of oral cancer	Non cancer cases from hospital	*Naswar* (Non Chew)	Age, sex, and MAS variables	Age, and sex	5

*Abbreviations*: *NOS* Newcastle-Ottawa Scale, *SLT* smokeless tobacco, *MAS* minimum Adjustment Set

### SLT use and OCC risk in men and women respectively

**Fig. 2:**
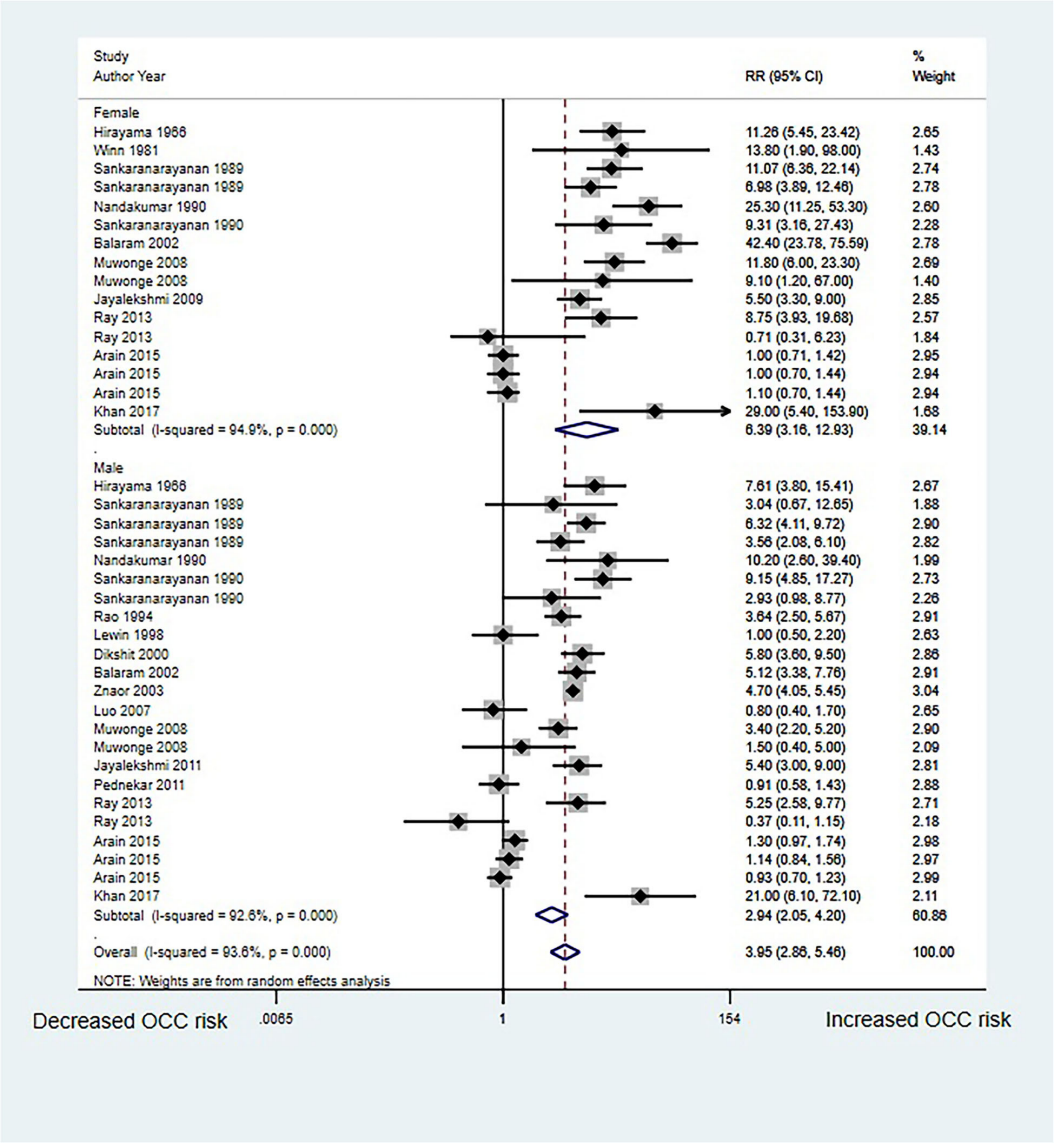
Association of SLT use with OCC risk in men and women

Sixteen studies in men and 11 studies in women reported the association between SLT use and OCC risk. We noted that SLT use was associated with an increased risk of OCC in both men (RR, 2.94; 95%CI, 2.05–4.20; *P* < 0.001) and women (RR, 6.39; 95%C, 3.16–12.93; *P* < 0.001) and women had a much higher risk than did men (Fig. 2). Significant heterogeneity was observed for both studies conducted with men (*I*^*2*^ = 92.6%; *P* < 0.001) and women (*I*^*2*^ = 94.9%; *P* < 0.001).

### Sex difference in the relationship between SLT use and OCC risk

**Fig. 3:**
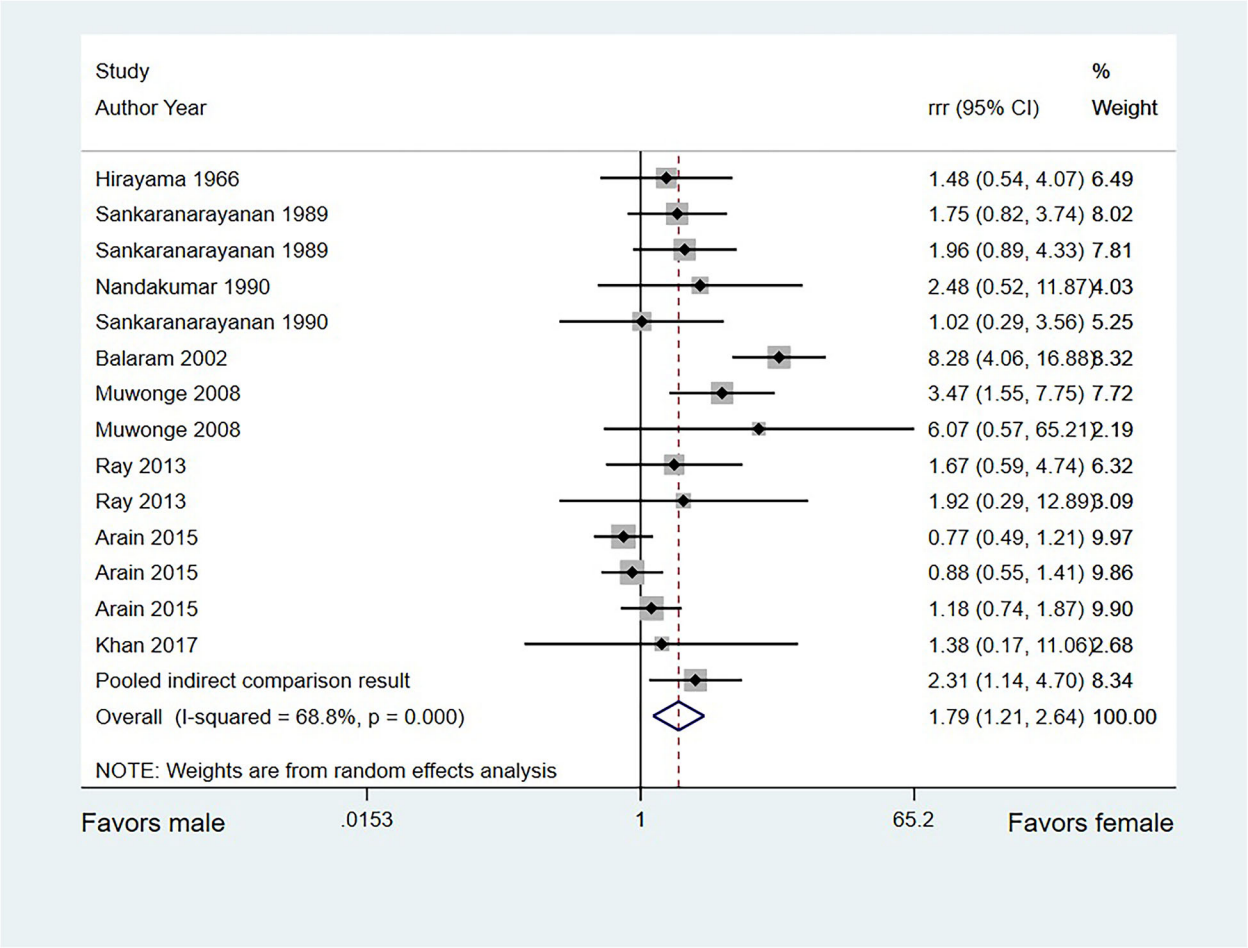
Sex-based difference in the relationship between SLT use and OCC risk in the whole cohort

**Fig. 4:**
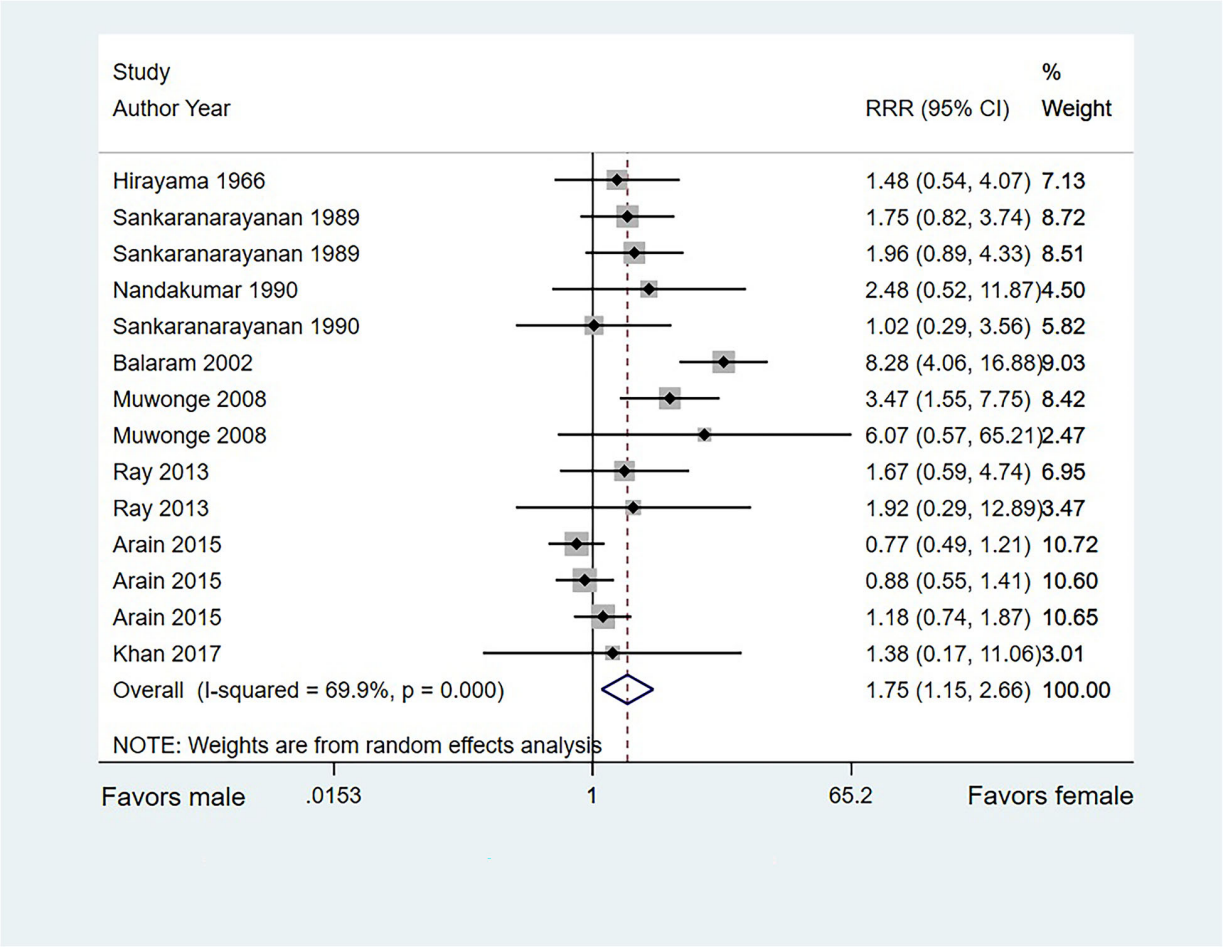
Sex difference in the relationship between SLT use and OCC risk based on direct comparison results

A total of 10 studies directly compared the sex-based difference in OCC risk associated with SLT use, and the remaining 9 studies only reported the relationship between SLT use and OCC risk in a single-sex population. The overall pooled RRR suggested that SLT use in women was associated with an increased risk of OCC compared with that in men (RRR, 1.79; 95%CI, 1.21–2.64; *P* = 0.003; Fig. 3). Significant heterogeneity was found across the included studies (*I*^*2*^ = 68.8%; *P* < 0.001). A significant difference was also found in the pooled RRR of indirect comparisons (RRR, 2.31; 95%CI, 1.14–4.70; *P* = 0.021). After excluding indirect comparison results, the conclusion was stable and not altered (RRR, 1.75; 95%CI, 1.15–2.66; *P* = 0.008; Fig. 4).

Subgroup analysis suggested significant sex-based difference only in individuals who received chewed smokeless products, regardless of the control definition. A pooled analysis of studies reporting on adjusted effect estimates, using matched controls, and with high quality confirmed the notably higher risk of OCC in women than that in men (Table 2).

**Table 2:** Subgroup analyses for sex-based difference in the association between smokeless tobacco use and oral cavity cancer risk

Factors	Groups	Number of cohorts	RRR and 95% CI	*P* value	*I*^*2*^ (%)
Type of smokeless product	Chew	9	2.09 (1.17–3.75)	**0.013**	78.7
	Non-chew	3	0.94 (0.60–1.47)	0.774	0.0
	Mixed	2	1.51 (0.79–2.90)	0.210	0.0
Control definition	Hospital-based	12	1.58 (1.03–2.44)	**0.037**	70.0
	Population-based	2	3.68 (1.72–7.87)	**0.001**	0.0
Confounders adjusted	Yes	12	1.75 (1.10–2.79)	**0.018**	74.3
	No	2	1.72 (0.74–4.03)	0.209	0.0
Matching of control	Matched	12	1.83 (1.13–2.96)	**0.013**	74.2
	Unmatched	2	1.51 (0.79–2.90)	0.210	0.0
Study quality	High	3	2.72 (1.57–4.71)	**< 0.001**	0.0
	Moderate	11	1.55 (0.97–2.48)	0.066	72.0

*P*-values < 0.05 were marked in bold

### Publication bias

**Fig. 5:**
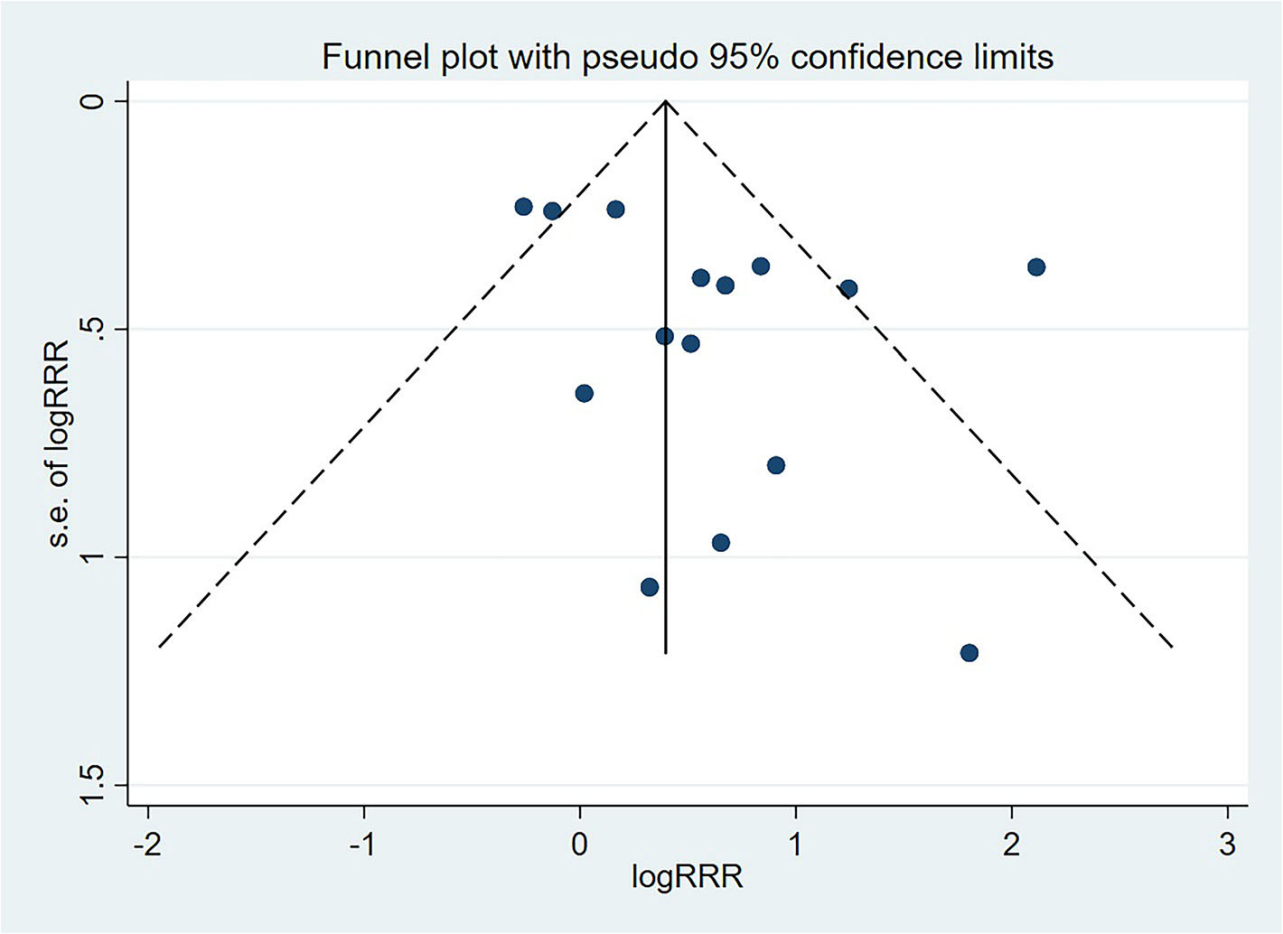
Funnel plot for the sex-based difference of SLT use with OCC risk

Potential publication bias for sex-based difference in the association between SLT use and OCC risk was observed by reviewing a funnel plot (Fig. 5). However, no significant publication bias was detected through Egger (*P* = 0.123) or Begg test (*P* = 0.488).

## Discussion

Our study provided both direct and indirect comparisons between SLT use and OCC risk for the first time. We found that SLT use was a strong and independent risk factor for OCC in both men and women. SLT use in women was associated with an increased risk of OCC compared to that in men, irrespective of whether the whole cohort was considered, or only direct comparison results were considered. Subgroup analysis indicated that the sex-based differences were more evident in populations receiving chewed SLT products and in studies with high-quality designs.

The pooled result of this study found that the OCC risk related to SLT use in women was significantly higher than that in men based on direct or indirect comparison results. However, among the studies included in the direct comparison, no significant differences were found between men and women, and only 2 studies [38, 41] observed a significant sex-based difference of the relationship between SLT use and OCC risk. Muwonge et al. [41] found significant sex-based differences in OCC risk in individuals who use *pan* and tobacco (chewed), while no significant sex-based difference was detected in those who use areca nut/lime and tobacco (chewed). Moreover, Balaram et al. [38] found that the OCC risk in women was significantly higher than that in men who used *pan* with tobacco (chewed). This could be explained by the differences in behavior and methodological issues, including the levels of background smoking and drinking, smaller sample size and lower event rates in women. Most studies had adjusted for confounding variables including age, alcohol, smoking, religion, education, and residential area and matched the controls with cases. However, the possible impact of these confounders was difficult to determine in the present analysis. Therefore, the reliable of pooled results needs further verification by fully adjusting for potential confounders in large-scale populations with long-term follow-up.

Subgroup analysis suggested that significant sex-based differences in SLT-associated OCC risk were found only in individuals receiving chewed smokeless products, regardless of the control definition used in the studies. This finding could probably be attributed to the chewing of SLT product containing betel nut, which was associated with a high risk of OCC [48], and the different types of SLT product in specific regions, which could affect OCC progression. Furthermore, studies with adjusted effect estimates, matched controls, and high quality significantly correlated with evidence level, balance of characteristics in the case and control groups, and stability of individual results, respectively. Subgroup analysis of high-quality studies further proved the obvious sex-based difference in the association between SLT use and OCC risk.

Numerous studies [12–14] have already illustrated the harmful effects of SLT use on the risk of cancer in oral, pharyngeal, laryngeal, and esophageal cancers. A meta-analysis conducted by Weitkunat et al. [12] that included 32 epidemiological studies before the 1980s and case-control studies with hospital-based controls found SLT use in Americans or Europeans caused a minor increase in the risk of OCC, with the increase being more pronounced in women than that in men. Sinha et al. [13] conducted a meta-analysis of 25 studies and performed a sex-wise subgroup analysis of OCC risk in SLT users. Their study revealed that women had a higher risk of OCC than did men (OR = 12.0 vs. 5.16). In a meta-analysis by Asthana et al. [14], a significant positive relationship was observed between SLT use and OCC risk, especially in women and users from Southeast Asian and Eastern Mediterranean regions. However, estimates for the sex-based difference between SLT use and OCC risk based on direct comparisons were not given in the previous meta-analysis [12–14]. Our systematic review and meta-analysis included recent publications and specifically assessed sex-based difference for the association between SLT use and OCC risk. Our study confirmed a higher OCC risk in female SLT users than in male users.

The strengths of this study should be highlighted: (1) this study is the first to estimate the sex-based difference in the relationship between SLT use and OCC risk based on whole cohort and direct comparison results; (2) the pooled result of this study was based on a large number of individuals and would be more robust than those of any individual study; and (3) the results of the subgroup analysis based on the study or participants’ characteristics could help screen the SLT users at high risk for OCC.

However, the limitations of this study should be acknowledged as well: (1) this meta-analysis was based on both prospective and retrospective observational studies, which caused inevitable selection and recall bias; (2) several included studies only provided crude effect estimates, which could bias the pooled effect estimate; (3) subgroup analysis stratified by specific SLT products was not conducted owing to the small number of included studies; and (4) potential publication bias was inevitable because of the unavailability of unpublished data.

## Conclusions

This study found that SLT use was associated with a higher risk of OCC in women than in men. Further large-scale prospective cohort studies should be conducted to verify sex-based difference in specific smokeless products.

## Data Availability

The datasets used and analyzed in the current study are available from the corresponding author upon reasonable request.

## Abbreviations

OCC: Oral cavity cancer
SLT: Smokeless tobacco
NOS: The Newcastle-Ottawa scale
RR: Relative risk
RRR: Ratio of relative risk
CI: Confidence interval
